# Genome-Scale Metabolic Model Driven Design of a Defined Medium for *Campylobacter jejuni* M1cam

**DOI:** 10.3389/fmicb.2020.01072

**Published:** 2020-06-19

**Authors:** Noemi Tejera, Lisa Crossman, Bruce Pearson, Emily Stoakes, Fauzy Nasher, Bilal Djeghout, Mark Poolman, John Wain, Dipali Singh

**Affiliations:** ^1^Microbes in Food Chain, Quadram Institute Biosciences, Norwich Research Park, Norwich, United Kingdom; ^2^SequenceAnalysis.co.uk, NRP Innovation Centre, Norwich, United Kingdom; ^3^University of East Anglia, Norwich, United Kingdom; ^4^Department of Veterinary Medicine, University of Cambridge, Cambridge, United Kingdom; ^5^London School of Hygiene and Tropical Medicine, University of London, London, United Kingdom; ^6^Cell Systems Modelling Group, Oxford Brookes University, Oxford, United Kingdom

**Keywords:** *Campylobacter jejuni*, genome-scale metabolic model, linear programming, defined growth media, metabolism, auxotrophy, metabolic network

## Abstract

*Campylobacter jejuni*, the most frequent cause of food-borne bacterial gastroenteritis, is a fastidious organism when grown in the laboratory. Oxygen is required for growth, despite the presence of the metabolic mechanism for anaerobic respiration. Amino acid auxotrophies are variably reported and energy metabolism can occur through several electron donor/acceptor combinations. Overall, the picture is one of a flexible, but vulnerable metabolism. To understand *Campylobacter* metabolism, we have constructed a fully curated, metabolic model for the reference organism M1 (our variant is M1cam) and validated it through laboratory experiments. Our results show that M1cam is auxotrophic for methionine, niacinamide, and pantothenate. There are complete biosynthesis pathways for all amino acids except methionine and it can produce energy, but not biomass, in the absence of oxygen. M1cam will grow in DMEM/F-12 defined media but not in the previously published *Campylobacter* specific defined media tested. Using the model, we identified potential auxotrophies and substrates that may improve growth. With this information, we designed simple defined media containing inorganic salts, the auxotrophic substrates, L-methionine, niacinamide, and pantothenate, pyruvate and additional amino acids L-cysteine, L-serine, and L-glutamine for growth enhancement. Our defined media supports a 1.75-fold higher growth rate than Brucella broth after 48 h at 37°C and sustains the growth of other *Campylobacter jejuni* strains. This media can be used to design reproducible assays that can help in better understanding the adaptation, stress resistance, and the virulence mechanisms of this pathogen. We have shown that with a well-curated metabolic model it is possible to design a media to grow this fastidious organism. This has implications for the investigation of new *Campylobacter* species defined through metagenomics, such as *C. infans*.

## 1. Introduction

### 1.1. Background

*Campylobacter*, the leading cause of acute bacterial gastroenteritis, is Gram-negative, microaerophilic, spiral-shaped, highly motile bacteria (Debruyne et al., 2008; Lastovica et al., 2014). The epidemiology in low to middle income countries is poorly studied but appears to be different from that in high-income countries (Platts-Mills and Kosek, 2014). Acquisition in high income countries is mainly through ingestion of contaminated poultry, milk, or water (Altekruse et al., 1998; Butzler, 2004; Ruiz-Palacios, 2007; Westrell et al., 2009). The genus *Campylobacter* can be divided into 28 species (Wilkinson et al., 2018) (and further into sub-species), that may vary in their temperature, oxygen, and nutrient requirements for growth. The most common species associated with campylobacteriosis is *Campylobacter jejuni*, with *Campylobacter coli* reported in 10–25% of cases. The disease is economically important, with an estimated cost to the UK economy at around £50 million (Tam and O'Brien, 2016). Under reporting of cases (Gibbons et al., 2014) and failure to consider sequelae (Mangen et al., 2016), however, makes this an underestimate. Disease symptoms in humans may vary, but in the UK, they include diarrhea, fever, and abdominal pain. The sequelae however include colitis, reactive arthritis, and Miller-Fisher and Guillain-Barré syndromes (Jacobs et al., 1996; Blaser and Engberg, 2008). Along with an increase in reported incidence, and improving diagnosis, resistance to antibiotics is becoming a concern (Châtre et al., 2010; Deckert et al., 2010; Gormley et al., 2010). The diagnosis of campylobacteriosis has depended, until recently, on culture-based methods, but these methods may not grow all *Campylobacter* spp. causing human infections (Buss et al., 2019; Bian et al., 2020). An understanding of how to grow these “non-culturable” *Campylobacter* spp. may change our understanding of campylobacterosis, particularly in low income countries where the burden is high and predominantly occurs in children (Platts-Mills and Kosek, 2014). The conditions used in diagnostic laboratories vary, but commonly include a gas mix of 5–10% O_2_, 5–10% CO_2_ and 80–85% N_2_ (with some mixes also containing H_2_), a temperature of 37°C or 42°C, with complex media containing blood, peptone, meat, or yeast extract (Hsieh et al., 2018). These media are not chemically defined, and may vary from batch to batch, making comparisons between strains and the growth conditions used for isolation difficult. The use of recently developed commercial PCR assays have improved diagnosis, but new variants would not be detected. As metagenomic methods are not yet freely available, a more permissive culture media is required for comprehensive burden studies and pathogen discovery.

#### 1.1.1. Growth Culture

Until the introduction of selective agars (Skirrow, 1977; Lauwers et al., 1978), filtration techniques were used to isolate *Campylobacter* (Dekeyser et al., 1972; Butzler et al., 1973). An understanding of the microaerophilic nature of *Campylobacter* (Hoffman et al., 1979) improved the culture still further. The development of *Campylobacter* specific culture media (Corry et al., 1995; Davis and DiRita, 2008; Kim et al., 2016) now represents the state of the art, however, the availability of a permissive defined media for *Campylobacter* spp. remains elusive. Although defined media have been described (Dickgiesser and Czylwik, 1985; Tenover et al., 1985; Guccione et al., 2010; Alazzam et al., 2011), the use of complex, undefined media (Wright et al., 2009; Liu et al., 2012) persists, because of the need to culture a wide range of *Campylobacter* strains that demonstrate different auxotrophies (Dickgiesser and Czylwik, 1985; Tenover and Patton, 1987; Alazzam et al., 2011; Vorwerk et al., 2014). A method to investigate the ingredients required for a permissive media is clearly needed. One way to do this is to look at the metabolic requirements of campylobacter cells in terms of nutrient sources, biomass, and energy production.

#### 1.1.2. Campylobacter Metabolism

*Campylobacter* spp. have considerable flexibility for energy production in terms of electron acceptors and donors that can be used in the electron transport chain (ETC); in addition to the ability to utilize O_2_ as an electron acceptor, nitrate, nitrite, and fumarate have also been described as alternatives. *Campylobacter* spp. would therefore appear to have the potential to carry out anaerobic respiration (Sellars et al., 2002; Kelly, 2008), however, many strains do not grow in the absence of oxygen, even in the presence of alternative electron acceptors (Sellars et al., 2002). The reason for this oxygen dependency is not absolutely certain but is likely to be related to oxygen dependent DNA synthesis enzymes (Jordan and Reichard, 1998; Kelly, 2008). The ETC also has a high degree of flexibility in terms of electron donors, with pyruvate, H_2_, formate, and succinate having been reported to fulfil this role (Sellars et al., 2002; Kelly, 2008). In contrast to the ETC, other areas of central metabolism are quite limited, when compared with that of most other gut bacteria. In particular, they lack transporters for many small carbohydrates, with two essential enzymes in the upper limb of the Embden–Meyerhof–Parnas pathway (glucokinase and phosphofructokinase) being absent. Most strains lack the Entner-Doudoroff pathway, and the oxidative steps of the oxidative pentose phosphate pathway are also absent (although the non-oxidative branch is present Velayudhan and Kelly, 2002; Line et al., 2010). The absence of carbohydrate utilization pathways suggests that amino acids and tricarboxylic acid (TCA) cycle intermediates act as the primary nutrient sources for *Campylobacter jejuni*(Westfall et al., 1986; Velayudhan et al., 2004; Del Rocio Leon-Kempis et al., 2006; Stahl et al., 2012; Hofreuter, 2014), but detailed knowledge as to how such substrates may be utilized, remains limited. Furthermore, the ability of this organism to survive right through the food chain, suggests it can utilize a variety of metabolic strategies. Our understanding of *Campylobacter* physiology, thus, remains limited, with most descriptions of *Campylobacter* physiology being based on a single isolate of *Campylobacter jejuni* NCTC11168 (Carrillo et al., 2004; Alazzam et al., 2011; Metris et al., 2011; Xu et al., 2015; van der Hooft et al., 2018).

### 1.2. Metabolic Modeling

Genome-scale metabolic models (GSMs) describe the metabolic interactions of an organism with its environment, based on a reaction network ultimately inferred from enzymes encoded by the genome. Their analyses enable an investigation of metabolic behavior of the whole system, rather than individual reactions and pathways, and can be used to identify mechanistic links between cellular genotype and metabolic phenotype.

**Genome-scale metabolic models overview**

Over the last two decades, GSMs have become a valuable tool to analyze cellular behavior under different biological conditions, to design drug targets, to investigate metabolic interactions in microbial communities, and to design defined growth media (reviewed in Liu et al., 2010; Zhang and Hua, 2016; Kim et al., 2017; Gu et al., 2019). GSMs can now be used to inform experimental design and to provide a rationale for experimental observations (Zampieri and Sauer, 2016; Villanova et al., 2017; van der Ark et al., 2018).

Construction of GSMs starts with the identification of enzymes and transport proteins from an annotated genome. Reactions catalyzed by enzymes thus identified, are further characterized by their stoichiometry and assumed (ir)reversibility. Such information can usually be obtained from a number of on-line databases including databases such as BioCyc (Caspi et al., 2015; Karp et al., 2017), MetaCyc (Caspi et al., 2013, 2017), KEGG (Ogata et al., 1999; Kanehisa and Goto, 2000; Kanehisa et al., 2006), BIGG (Schellenberger et al., 2009), SEED (DeJongh et al., 2007), and BRENDA (Barthelmes et al., 2007).

In addition to defining the properties of internal reactions, it is, for all practical purposes, necessary to identify the properties of transport processes, representing the consumption and production of compounds in the assumed environment. Compounds present in the environment are referred to as *external* (or sometimes *boundary*) metabolites, otherwise they are referred to as *internal* metabolites. Reactions that only involve internal metabolites are therefore internal reactions, while those which involve internal and external metabolites are transport reactions. Although gene annotation with regard to enzymes is usually well-defined, annotation of transporters tends to be less comprehensive. Experimental investigations involving growth on defined media thus proves a useful independent method to infer the presence of transport processes, as well as providing useful data for subsequent analyses of the model.

Despite the fundamental importance of online databases, any reconstruction based solely upon these is likely to exhibit a number of problems, the most common of which are: missing gene-protein-reaction (GPR) associations, inconsistent naming of metabolites, and reaction identifiers, incorrect reaction stoichiometries, and reversibility, which propagates to the draft model. Thus, in order to represent a realistic representation of a given organism, an initial draft model will require refinement and curation by the user. The curation process usually begins with correcting reaction stoichiometries and reversibility to ensure that the model follows the law of mass and energy conservation, followed by checking consistency of identifiers, and identifying missing reactions by “gap-filling.” Such gap-filling may often lead to an updated gene annotation. One area of metabolism that requires special attention is the electron transport chain, to ensure that translocated protons are correctly assigned to their location in the cell or the environment.

Usually, large-scale metabolic models such as GSMs, are analyzed using constraint based linear programming (LP) approaches (Fell and Small, 1986; Varma and Palsson, 1993, 1994), an optimization technique that assigns fluxes to reactions, assuming that the system is at steady state, according to some objective function, and subject to one or more defined constraints. Typical objective functions are either maximization of growth rate, or minimization of total flux in the system, and constraints are used to apply upper or lower limits on reactions, and commonly to specify the export of one or more product as defined.

#### 1.2.1. Aims of the Current Study

The aim of this study is to explore *Campylobacter* spp. nutrient requirements through the analysis of a newly created genome scale model of *Campylobacter jejuni* (M1Cam) with experimental validation. In doing so we define new, chemically defined, media compositions that will serve as a basis for further modeling and experimental studies of this organism.

## 2. Materials and Methods

### 2.1. Model Construction

**Figure 1:**
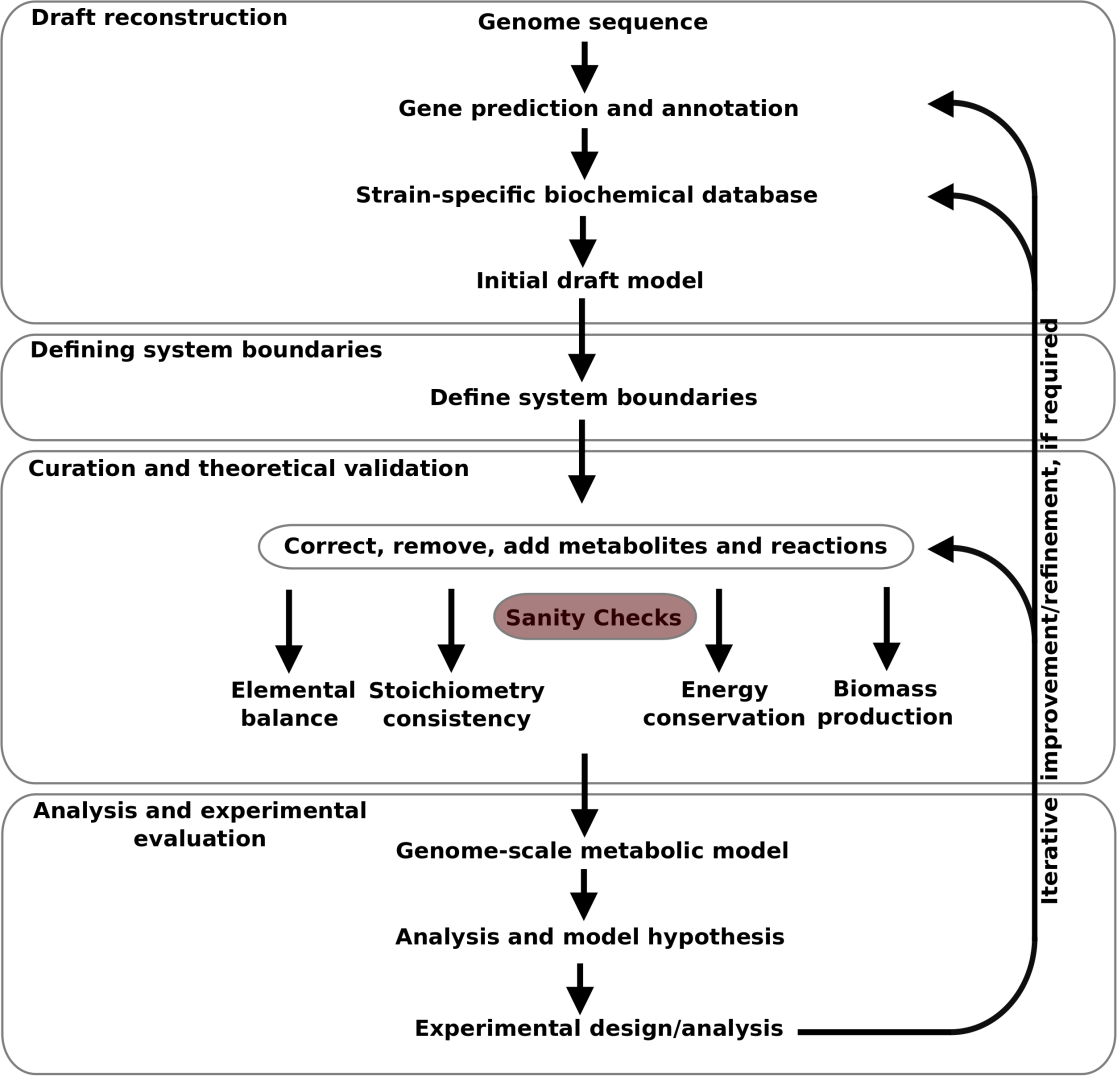
Pipeline for genome-scale metabolic model construction used in this study.

The model was developed in an iterative manner, as illustrated in Figure 1, from the available gene sequence of *Campylobacter jejuni* M1cam (https://www.ncbi.nlm.nih.gov/nuccore/CP012149). Genes were predicted and annotated, and used to construct a strain specific Pathway/Genome database (PGDB) for *Campylobacter jejuni* M1cam. This was used as the basis for the model which was structured in a modular fashion as described by Poolman et al. (2009), Poolman et al. (2013), Hartman et al. (2014), and Ahmad et al. (2017). It consists of:

1. A “top-level” module defining some basic properties and importing the other modules as described;
2. A “PGDB reactions” module with a set of reactions directly imported from the strain-specific PGDB which was subjected to revision as described in 2.1.3.
3. “Media transporter” module describing the import and export of substrates to/from the medium.
4. “Biomass transporter” module describing the export of biomass precursors and metabolites produced as side products of biomass synthesis for which there are no known degradation pathways, also called “sink transporters.”
5. The ETC module describing stoichiometries of all ETC reactions.
6. Additional reactions required for the generation of biomass precursors, not reported in the PGDB.

#### 2.1.1. Draft Reconstruction

Gene prediction and annotation was carried out using Prodigal (Hyatt et al., 2010) version 2.6.31 and Prokka (Seemann, 2014) version 1.142 respectively, with default cutoff scores.

The “Pathologic component” of Pathway Tools (Karp et al., 2002, 2015) version 23.0 and MetaCyc (Caspi et al., 2017) version 23.0 was used to generate the strain-specific Pathway/Genome databases (PGDB), from the annotated genome sequence. The pathway hole filler component (Green and Karp, 2004) of Pathway Tools was used to automatically fill pathway holes in PGDB. Pathways were inferred using the pathway prediction algorithm (Karp et al., 2011) with a default cutoff score of 0.15. Missing genes and GPR associations (see sections 3.1.1), were curated manually with reference to BLAST (Camacho et al., 2009) and the online databases KEGG (Ogata et al., 1999), Gene Ontology terms (Ashburner et al., 2000), BRENDA (Jeske et al., 2018), Pfam (El-Gebali et al., 2018), RHEA (Morgat et al., 2019), and String v11 accessed 2019 (Szklarczyk et al., 2018).

All the reactions from PGDB were extracted, using the ScrumPy (Poolman, 2006), metabolic modeling package, version 1254, to populate the “PGDB reactions” module described above, and to generate the initial draft model.

#### 2.1.2. Defining System Boundaries

*Media transporters* were assigned for import of 33 organic substrates, out of 38 organic substrates in the DMEM/F-12 medium (Table S1). Transporters for the remaining five organic substrates, choline chloride, folic acid, lipoic acid, thymidine, and vitamin B12 (cobalamine) were not included because metabolic routes were not present for these metabolites in the PGDB. Transporters were assigned for exchange of the gases O_2_, CO_2_, H_2_, NH_4_ and H_2_S between the environment and the system and for the export of metabolic by-products, such as acetate, lactate, and succinate.

*Biomass transporters* were defined for the export of biomass components as individual components (amino acids, lipopolysacharides, peptidoglycan, nucleotides, vitamins, etc.), as described by Thiele et al. (2005) and Metris et al. (2011). Note that in contrast to these authors who represented biomass as a single lumped reaction, we assigned biomass transporters to individual biomass components, and subsequently set their relative fluxes to represent the observed biomass composition. There are five sink transporters added to the model for export of by-products from biomass synthesis [eg. adenosyl-4-methylthio-2-oxobutanoate produced during biotin synthesis (Metris et al., 2011)].

#### 2.1.3. Curation and Theoretical Validation

The atomic balance of individual reactions were determined and all the reactions in the model were balanced with respect to carbon (C), nitrogen (N), phosphorus (P), and sulfur (S), iron (Fe), sodium (Na), oxygen (O), proton (H), and charge. Metabolites with unknown atomic compositions or generic compounds and reactions involved with such metabolites were replaced, where possible, by balanced reactions with known atomic composition of metabolites. For example, reactions involved with generic compounds (e.g., Alcohols) were replaced with specific metabolites and the stoichiometric coefficient balanced on both sides of the reaction. Reactions involved with non-metabolic species (e.g., tRNA, RNA) were excluded from the model. Similarly, reactions were corrected for reversibility so that the model is not able to generate energy (in the form of ATP and NAD(P)H) in absence of a mass flux through the system. Thus, the model is ensured to follow the laws of mass and energy conservation, and is free from stoichiometric inconsistencies (Gevorgyan et al., 2008).

### 2.2. Model Analysis

#### 2.2.1. Linear Programming Formulation

Except where noted otherwise, model analysis was undertaken using appropriate variants of the linear program defined as:

(1) $$\begin{array}{l} \text{minimise}:\sum \left|\text{v}\right|\\ \text{subject to}:\left\{ \begin{array}{l} N.v=0\\ \;v_{i..j}{\text{= b}}_{i..j}\\ v_{k..l}\geq 0\;(\text{see below})\\ v_{\text{ATPase}}\text{ = A}\\ v_{Otx}\leq Otx_{max} \end{array}\right. \end{array}$$

Where **v** is the vector of all reaction fluxes and **N** is the stoichiometry matrix; the objective is to minimize the sum of all (absolute) flux values (including transporters), subject to the constraints: **Nv** = **0** (steady-state assumption), *v*_*i*..*j*_ = *b*_*i*..*j*_ defines flux in biomass transporters, as described in section 2.1.2, *v*_*k*..*l*_ defines fluxes in the reactions importing media components, as described in section 2.1.2, *v*_*ATPase*_ = *A* defines flux in a hypothetical ATPase reaction in order to account for a growth and non-growth associated maintenance cost and *v*_*Otx*_ ≤ *Otx*_*max*_ defines a maximum microaerophilic O_2_ consumption rate. *A* was assigned a value of 16 mmol/g DW/hr (Varma and Palsson, 1994), and *Otx*_*max*_ (= 5 mmol/g DW/hr) (Metris et al., 2011).

#### 2.2.2. Identification of Substrate Auxotrophies

Substrate auxotrophies were determined, using the model, by repeatedly attempting to solve Equation (1) with individual media transporters (*v*_*k*..*l*_) set to zero, one at a time. Failure to obtain a solution was taken as demonstrating auxotrophy with respect to the component whose transporter was thus constrained.

### 2.3. Model Curation and Refinement From Experimental Observation

Although *Campylobacter* spp. was successfully cultured in the DMEM/F-12 medium (section 2.4), the initial model could not account for the production of all biomass components from these nutrients. These components were identified by modifying Equation (1) to account for the production of a single biomass component, sequentially. Additional reactions were then identified by their presence in other *Campylobacter* spp. specific biochemical databases in Biocyc and KEGG, literature surveys, and with reference to BLAST (Camacho et al., 2009). Once identified, these reactions were used to populate the “Additional Reactions” module described above.

Initial model analysis suggested that biotin and asparagine were essential nutrients, although this was found not to be the case in the experimental investigation. This discrepancy was due to gaps in the biosynthesis pathways of biotin and asparagine. Gap-filling reactions were identified, based on a literature study on biotin synthesis (Stok and Voss, 2000; Manandhar and Cronan, 2017) and BlastP analysis, and were added to the “Additional Reactions” module as above.

Conversely, initial model analysis suggested that niacinamide was not an essential nutrient, although subsequent experimental investigation showed that this was not the case. In order to identify spurious reactions in the model responsible for this heterotrophy, Equation (1) was modified to represent *production* of niacinamide, utilizing the other media components. Reactions in the resulting solution for which no GPR could be identified were removed, thus bringing model behavior into alignment with our experimentally observed behavior.

### 2.4. Experimental Conditions

#### 2.4.1. Materials

Brucella broth was purchased from Sigma-Aldrich (Sigma-Aldrich, Poole, UK) and Brucella agar from BD (Becton, Dickinson U.K. Limited). Gibco Dulbecco's Modified Eagle Medium/Nutrient Mixture F-12 (DMEM/F-12) media with no added phenol red, was purchased from Thermo Fisher Scientific (Thermo Fisher Scientific, Darmstadt, Germany). All chemicals were of commercial analytical grade and obtained from Sigma-Aldrich. All solutions, except amino acids and vitamins, were made every week, sterile filtered (0.22 μM, Fisher-Scientific, UK), and kept at −4°C until needed. Amino acids and vitamins were prepared immediately before use.

#### 2.4.2. Bacterial Strains Used

*Campylobacter jejuni* M1 has a published genome sequence (Friis et al., 2010) and is a well-studied laboratory model organism from a well-documented case of direct transmission between poultry and humans. The M1 used in this study was obtained from the Department of Veterinary Medicine, Cambridge University, and annotated as M1cam (de Vries et al., 2015), an “M1” strain which differs from the M1 reference strain (published by Friis et al., 2010) by 50 SNPs (insertions/deletions and true SNPs) (de Vries et al., 2015).

The other 7 strains, selected from different clades, according to Champion et al. (2005) and Stabler et al. (2013), were tested for their ability to grow in the final defined medium. These were: 81116 (NCTC11828), Calf 3, 11168H, 81-176, 12912, RM1221, and M1, obtained from the London School of Hygiene and Tropical Medicine. Strains were stored at −80°C using Protect - Multipurpose Microorganism Preservation System vials (Technical Service Consultants Ltd, Lancashire, UK) or Microbank^*TM*^ cryotubes (Pro-Lab Diagnostics, Merseyside, UK).

#### 2.4.3. Culture Conditions

All strains were grown under microaerophilic conditions (10% O_2_ [v/v], 5% CO_2_ [v/v], 85% N_2_ [v/v]), using an anaerobic cabinet and gas supply (BOC Ltd, Surrey, UK), on Brucella broth agar plates, for 48 h at 37°C, except those tested in the Department of Veterinary Medicine, Cambridge University, which were grown at 42°C using a gas mix of hydrogen (5% O_2_ [v/v], 5% H_2_ [v/v], 5% CO_2_ [v/v], 85% N_2_ [v/v]), also using an anaerobic cabinet and gas supply (BOC Ltd, Surrey, UK).

All *Campylobacter jejuni* strains were harvested from agar plates and inoculated with a starting optical density (OD) OD600 of approximately 0.004 in Brucella broth, incubated with shaking (180 rpm) using the same temperature and gas supply indicated previously, to an OD600nm of 0.6 (approximately 2.8 × 10^9^ CFU/ml). Cultures with OD600 different to 0.6 were standardized to that value using sterile PBS. After 20 h, 1.7 mL of this culture was collected, washed twice and resuspended in 1.7 mL of sterile PBS. Each one of the media tested in our study was inoculated in triplicate at 1% (100 μL in 10 mL of media), using *Campylobacter jejuni* washed cells. Cultures were then incubated microaerobically for 48 h with shaking (180 rpm), under the same temperature and gas conditions stated above, and growth was assessed daily, measuring OD600 in triplicate of each of the technical replicates, using a plate reader (FLUOstar® Omega, BMG Labtech Ltd, Aylesbury, UK). For this purpose, cultures were sampled in a class II cabinet and returned immediately to the anaerobic cabinet. All experiments performed in this study were repeated three times, each time using a new starting culture, prepared from glycerol stock, and with freshly made simple defined media.

#### 2.4.4. Checks for Contamination

To confirm the identity of the experimental organisms, typical cellular morphology was checked using x1000 bright field microscopy of carbol fuchsin stained cultures and whole genome sequencing (WGS) was performed on selected isolates. *Campylobacter jejuni* M1cam genomic DNA for WGS was extracted using Maxwell® RSC Cultured Cells DNA Kit. DNA extracts were converted into a Nextera library for sequencing on an Illumina NextSeq 500 platform and genomes were assembled using Velvet *de-novo* genomic assembler and then subjected to multi-locus sequence typing (MLST).

#### 2.4.5. Media Design and Confirmation of Auxotrophies

A modification of DMEM/F-12, not including glucose, was used as the starting point to develop our defined media for *Campylobacter jejuni* M1cam (Table S1). The inorganic substrates in our media were kept in the same concentrations present in the original DMEM/F-12 media formulation. To achieve a simple defined media, non-essential substrates identified by the model analysis, were gradually removed from the media, and growth, measured as OD, was compared to that of growth in Brucella broth (0.58 ± 0.06 at 24 h) and synthetic DMEM/F-12 media (0.38 ± 0.01 at 24 h). Removal of substrates in media design were compensated for the net amount of N and S, by increasing the concentration of remaining substrates proportionally to ensure that the total N and S was equal or higher than the original formulation.

Substrate auxotrophy was confirmed when removal of substrate could not support growth of M1cam. Growth was monitored over longer incubation times (up to 144 h) to confirm auxotrophy of the compounds tested. If no growth was obtained, experiments were repeated, to confirm results.

#### 2.4.6. Media Testing

The media to be tested was made fresh on the day of the inoculation. After addition of all components and before adjusting the final volume to 100 mL using purified water, the pH of each of the minimal media tested in this study was measured using a pH meter [LAQUAtwin pH Meter PH-22 (HORIBA Advanced Techno Co., Ltd., Kyoto, Japan)] and adjusted to pH 7 using 1M NaOH or 1M HCl solutions. To maintain sterility, work was carried out in a class II microbiological safety cabinet, and 10 mL of media was filtered into each of the four 50 mL sterile cell culture flasks (Sigma-Aldrich, Poole, UK), closed with their filter caps and kept at 37°C until inoculation. Maintaining media at 37°C until inoculation proved to be very important to obtain reproducible results. Finally, in order to compare our results with previously published defined media, we also tested growth of *Campylobacter jejuni* M1cam in the defined media given by Guccione et al. (2010) and Alazzam et al. (2011).

## 3. Results

### 3.1. Model Building and Analysis

#### 3.1.1. Pathway/Genome Databases

The automatically generated *Campylobacter jejuni* M1cam PGDB comprised of 228 pathways with a total 1,366 reactions, including 22 transport reactions, and 1,009 compounds. There were 291 reactions without any assigned gene and were inferred from pathway prediction algorithm. This also included reactions that were essential for biomass productions in the model. There were also genes that encoded metabolic enzymes but were not associated with reactions. The reason for the missing GPR association was mainly: (i) information necessary to attribute a particular reaction to specific gene was missing in the annotated file (ii) though the gene product had been annotated it was not detected by the Pathologic software. Reactions, particularly those inferred as essential for biomass production, without any gene association were revisited to establish GPR relation (see 2.1.1) where possible. Table S2 lists GPR relationships that have been curated in the PGDB. Curated PGDB, after being re-inferred using pathway prediction algorithm, comprises a total 262 pathways, 1,581 reactions, and 1,104 compounds (summarized in Table 1 along with other prokaryotic PGDBs) and is available from Pathway Tools Registry and https://data.quadram.ac.uk/dipali.singh/CJM1cam_DB/.

**Table 1:** Main content of *C. jejuni* M1cam Pathway/Genome database along with other prokaryotic databases.

**Content/Strains**	***Campylobacter jejuni***	***Campylobacter jejuni***	***H. pylori***	***P. aeruginosa***	***Escherichia coli***
	**M1cam**	**M1**	**26695**	**PAO1**	**K-12 (MG1655)**
PGDB curation level	Tier 2	Tier 3	Tier 2	Tier 3	Tier 1
Total genes	1,679	1,675	1,610	5,677	4,501
Total pathways	262	228	152	405	433
Total reactions	1,581	1,005	970	1,833	2,852
Total compounds	1,104	746	708	1,285	2,965

#### 3.1.2. Model Properties

The curated model consists of 994 reactions. The number is less than that of the initial draft model generated from the PGDB due to removal of reactions involved with non-metabolic species and those of unknown atomic composition, as described in 2.1.3. 76% of reactions in the model have gene associations, 6% are spontaneous, while the remainder are inferred by the gap filling algorithm or added after careful curation. The model includes 115 transporters, 968 internal metabolites and 93 external metabolites, summarized in Table 2.

**Table 2:** Summary of genome-scale metabolic model of *C. jejuni* M1cam.

Total no. of reactions		**994**
Total no. of transporters		115
Total no. of metabolites		1,061
Reactions	Spontaneous reactions	56
	With gene association	761
	Without gene association	170
	Added reactions	7
Transporters	media	41
	Exchange and by-products	18
	Biomass	51
	Sink	5
Metabolites	Internal metabolites	968
	External metabolites	93

#### 3.1.3. Model Analysis—Biomass Production

The solution to Equation (1), with DMEM/F-12 media, has a total of 326 reactions, including transporters. There are 165 essential for biomass production: removal of any one of these results in Equation (1) having no feasible solution. The solution has no net import flux of amino acids, leucine, arginine, histidine, valine, asparagine, phenylalanine, alanine, threonine, tyrosine, tryptophan, and lysine (i.e., flux in these amino acid media transporters were equal to flux in their respective biomass transporters). There was, however, notable net import of pyruvate, as a carbon source, and the amino acids, aspartate, serine, glutamine, and proline; Acetate, succinate, NH_4_ and CO_2_ were among the excreted by-products. The model is able to account for ATP generation under anaerobic conditions through oxidative phosphorylation, using nitrate or fumarate electron acceptors. However, it is not able to generate biomass in the absence of O_2_.

#### 3.1.4. Model Analysis—Identification of Substrate Auxotrophy

Out of the 33 organic substrates tested in the model, 30 substrates were identified as non-essential. The remaining three substrates, *methionine, pantothenate* and *niacinamide* (after the curation based on experimental observation in 2.3) were identified as auxotrophic. The pathways for the *de-novo* biosynthesis of these metabolites were absent in the PGDB and thus the model. These included homoserine O-succinyltransferase and methionine synthase from the methionine biosynthesis pathway, 3-methyl-2-oxobutanoate hydroxymethyltrasferase, pantothenate synthetase, and 2-oxopantoate reductase from the pantothenate biosynthesis pathway, and nicotinate-nucleotide diphosphorylase and quinolinate synthetase from the niacinamide biosynthesis pathway.

#### 3.1.5. Model Analysis—Specification of Minimal Nutrient Requirements

The model analysis suggests that growth can be supported with a medium containing only pyruvate, methionine, pantothenate, and niacinamide (in addition to inorganic salts). Furthermore, the fact that aspartate, serine, glutamine, and proline were imported in excess, relative to that required by the biomass composition, suggests that the presence of these compounds in the media would enhance growth.

### 3.2. Experimental Analysis

#### 3.2.1. Media Design and Confirmation of Auxotrophies

A simplified defined media containing pyruvate, four amino acids (methionine, cysteine, serine and glutamine or glutamate), pantothenate, and niacinamide (along with inorganic salts) supported growth of *C. jejuni* M1cam. The removal of *methionine, pantothenate* and *niacinamide* from the DMEM/F-12 media (Table S1) resulted in no growth of M1cam, thus, confirming auxotrophies predicted by the model analysis.

As part of the media development, the concentration of sodium pyruvate, as a carbon source, was increased from 0.5 to 10 mM. In addition, the concentrations of auxotrophic substrates, niacinamide and pantothenate, were optimized to 33.1 and 9.4 μM, respectively (Table 3). The inorganic substrates were kept identical to those in DMEM/F-12 media. Amino acids cysteine, serine and glutamine or glutamate, although not essential for growth, had a significant growth promoting effect i.e., removal of any of these substrates from the media decreased the growth compared to DMEM/F-12 media. In contrast, removal of any other amino acids or vitamins did not have any significant effect on growth as compared to DMEM/F-12 media.

**Table 3:** Defined media composition designed in this study.

**Substrates**	DMEM/F-12^*^	**MM1**	**MM2**	**MM3**	**MM4**
**Auxotropic**					
Methionine	115.7 μM	4 mM	4mM	8 mM	6.8 mM
Pantothenate	4.7 μM	9.4 μM	9.4 μM	9.4μM	9.4 μM
Niacinamide	16.6 μM	33.1 μM	33.1 μM	33.1 μM	33.1 μM
**Carbon source**					
Pyruvate	0.5 mM	10 mM	10 mM	10 mM	10 mM
**Growth-improving**					
Cysteine·HCl	100 μM	4 mM	4 mM	–	4 mM
Serine	250 μM	10 mM	10 mM	10 mM	–
Glutamine	2.5 mM	10 mM	–	10 mM	13.6
Glutamate	50 μM	–	20 mM	–	–
**Inorganic salts**	same as DMEM/F-12 (Table S1)				
**Growth (OD 600)**					
24 h	0.38 ± 0.01	0.31 ± 0.05	0.14 ± 0.03	0.02 ± 0.01	0.15 ± 0.01
48 h	0.34 ± 0.04	1.05 ± 0.06	1.07 ± 0.09	0.41 ± 0.04	0.57 ± 0.01

*OD results expressed as mean ± stdev (n = 3)*.

* *Table S1 for all the components of DMEM/F-12*.

Thus, four simple defined media compositions were defined, where all four included the auxotrophic substrates, methionine, pantothenate, and niacinamide along with pyruvate as a carbon source, and with different combinations of non-essential but growth-improving organic substrates, presented in Table 3.

The best results, in terms of growth, were obtained when cysteine, serine, and glutamine or glutamate where present (MM1 and MM2 in Table 3). Individual removal of cysteine and serine from the media decreased growth to 0.41 ± 0.04 and 0.57 ± 0.01 at 48 h, respectively (MM3 and MM4 in Table 3). Glutamate and glutamine were exchangeable, having similar effects on growth (MM1 and MM2 in Table 3). However, removal of both substrates at the same time resulted in around 4.5 times lower OD (0.24±0.03 at 48 h, data not shown) when compared to MM1 and MM2. Providing only the essential auxotrophic substrates, i.e., methionine, pantothenate, and niacinamide, together with pyruvate as a carbon source and inorganic compounds, did not support growth (0.05 ± 0.02, data not shown). This confirmed the impact of each of the growth promoting amino acids in culturing *Campylobacter jejuni* M1cam. Although each of MM1 and MM2 had a similar effect on growth of *Campylobacter jejuni* M1cam, MM1 was selected as final defined media, as it had a higher starting pH and required less NaOH to reach pH 7.

Therefore, this media was tested for growth of 7 additional *Campylobacter jejuni* strains, with the experiments being performed in two different laboratories, the Department of Veterinary Medicine, Cambridge University and the London School of Hygiene and Tropical Medicine. All isolates tested in this study grew in the designed defined media MM1 by 48 h, using the conditions of growth as defined by each laboratory, resulting in a range of OD600 from 0.23 to 1.05 (Table 4). By 48 h, all strains had reached a plateau and the replicates were stable. The variation in growth was probably caused by different growth conditions and the genetics of the strains.

**Table 4:** Growth of different *C. jejuni* strains in the defined media MM1, designed in this study.

**Labs and**		**Standardized**	**OD600**	
**Culture conditions**	**Strains**	**Inoculum (cfu/ml)**	**24 h**	**48 h**
**QIB^a^**				
Temperature: 37°C	M1cam (ST 137)	1.73e+09	0.31 ± 0.05	1.05 ± 0.06
Gas mix:	81116 (ST 283)	1.57e+09	0.17 ± 0.10	0.86 ± 0.11
10% O_2_, 5% CO_2_, 85% N_2_				
**Cambridge^b^**				
Temperature: 42°C	M1cam (ST 137)	7.63e+07	0.09 ± 0.04	0.75 ± 0.04
Gas mix:	calf 3 (ST 514)	4.28e+07	1.01 ± 0.05	1.11 ± 0.10
5% O_2_, 5% CO_2_, 5% H_2_,85% N_2_				
**London^c^**				
	11168H (ST 43)	1.07e+09	0.18 ± 0.12	0.23 ± 0.09
Temperature: 37°	81–176 (ST 604)	2.05e+09	0.13 ± 0.02	0.65 ± 0.10
Gas mix: 10% O_2_, 5% CO_2_, 85% N_2_	12912 (HS 50)	8.47e+07	0.34 ± 0.02	0.84 ± 0.12
	M1 (ST 137)	6.67e+08	0.07 ± 0.01	0.53 ± 0.12
	RM1221 (ST 354)	1.24e+08	0.36 ± 0.04	0.50 ± 0.07

*Standardized inoculum (OD = 0.6) colonies formation units/mL (cfu/mL) is expressed as average of replicates (n = 9). OD results expressed as mean ± stdev (n = 9). ST: sequence type*.

a *Quadram Institute Biosciences, UK*.

b *Department of Veterinary Medicine, University of Cambridge, UK*.

c *London School of Hygiene and Tropical Medicine, University of London, UK*.

#### 3.2.2. Testing Previously Published *Campylobacter* Specific Growth Media

We tested growth of M1cam on the previously published defined media by Guccione et al. (2010) and Alazzam et al. (2011) (MCLMAN media). Both formulations did not support growth of M1cam. MCLMAN media could support growth only after the addition of pantothenate, which was confirmed as an auxotrophic substrate for M1cam in our study. The OD at 600 nm of this culture (0.33 ± 0.03, data not shown) after 48 h of incubation, however, was still lower than the growth observed in our defined media (1.05 ± 0.06).

## 4. Discussion and Conclusion

In this study, we have constructed, a well-curated, strain-specific GSM of *Campylobacter jejuni* M1cam. We have examined the GSM to investigate the *de-novo* biosynthesis ability and substrate auxotrophy of the strain. We have experimentally validated the model results and curated the GSM for missing GPR associations or over-predicted reactions. A metabolic model guided the design of a simple defined media for the growth of *Campylobacter jejuni*. Additionally, we have shown that 8 diverse strains of *Campylobacter jejuni* are able to grow in the defined simple media.

### 4.1. Model Analysis

Our analysis showed that when all amino acids are available, leucine, arginine, histidine, valine, asparagine, phenylalanine, alanine, threonine, tyrosine, tryptophan, and lysine were imported at a rate equal to their demand for protein synthesis. However, the network described by the model has the ability for the *de novo* synthesis of these amino acids, as a solution to Equation 1 could still be found when their respective uptake transporters were blocked.

In contrast, aspartate, proline, serine, and glutamine were all taken up at a rate above that required for protein synthesis. It is worth noting that, without any constraint on media uptake, the model prefers uptake of aspartate, proline, serine, and glutamine, which are preferred by *Campylobacter jejuni* and are the most common amino acids found in chicken excreta (Parsons, 1984). In addition, the metabolic profile of *Campylobacter jejuni* liquid cultures' supernatant has shown significant depletion of these four amino acids from nutrient rich medium, reviewed in Hofreuter (2014).

Among all amino acids, serine has been most widely reported to be preferred by *Campylobacter jejuni*(Guccione et al., 2008; Wright et al., 2009; Gao et al., 2017). Glutamine utilization in *Campylobacter jejuni* is known to vary between strains: *Campylobacter jejuni* NCTC11168 has been reported not to utilize it (Hofreuter et al., 2008), while strains such as 81116 and 81-176 readily do (Hofreuter et al., 2006). Aspartate has been shown to be growth promoting under oxygen limitation (Guccione et al., 2008). Proline utilization has also been described at the stationary phase of *Campylobacter jejuni* growth, after the preferred nutrients become exhausted (Wright et al., 2009). These reports suggest that amino acid utilization varies between different strains of *Campylobacter jejuni*. It would therefore be necessary to build a model which represented that variation.

Whilst specific amino acids uptake from media remains to be tested experimentally for M1cam, similar experimental observations have been reported by Gao et al. (2017) where *Campylobacter jejuni* exhibits uptake capacity for amino acids such as arginine, cysteine, histidine, lysine, phenylalanine, or threonine that are directly incorporated into synthesized proteins rather than being catalyzed for energy or conversion to other intermediates.

Model analysis also reveals that *Campylobacter jejuni* M1cam has the ability for anaerobic respiration, however, it requires O_2_ for biomass. Although model observation is consistent with previous reports from Sellars et al. (2002), Kelly (2008), van der Stel et al. (2017), and van der Stel and Wösten (2019), this is a vast research topic in itself and details remain to be addressed experimentally.

### 4.2. Auxotrophic Substrates

The modeling and experimental results presented here, indicate that *Campylobacter jejuni* M1cam is auxotrophic for methionine, pantothenate, and niacinamide. In this study, we established that *Campylobacter jejuni* M1cam presents auxotrophy for methionine but not for cysteine. It can utilize methionine as a S source for cysteine biosynthesis as also reported in *Helicobacter pylorii*. The results presented here suggest that cysteine auxotrophy is strain dependent and not a conserved metabolic property in all *Campylobacter jejuni* strains as suggested by Vorwerk et al. (2014). On the contrary, we found that methionine auxotrophy is maintained in M1cam even under increased cysteine concentration. Cysteine, although not auxotrophic, is one of the growth improving components possibly because of its role in the *de novo* synthesis of proteins associated with FeS clusters, such as the serine dehydratase, pyruvate pyruvate:acceptor and oxoglutarate:acceptor oxidoreductases. Moreover, most *Campylobacter jejuni*, as a common feature of host associated epsilonproteobacteria, are unable to assimilate sulfate as the S source (Alazzam et al., 2011; Vorwerk et al., 2014) and to compensate for this limited anabolic capacity, it is reasonable to suggest that *Campylobacter* spp. retain auxotrophy (or prefer) S containing amino acids, methionine, and cysteine.

Auxotrophy for pantothenate, a precursor for CoA synthesis, is related to host specificity. The pantothenate biosynthesis gene (panB, panC, and panD) cluster lies in the hypervariable region (Pearson et al., 2003) and has been shown to be more frequently present in isolates from cattle compared to isolates from chickens (Sheppard et al., 2013). *Campylobacter jejuni* M1cam is the derivative of the human isolate *Campylobacter jejuni* M1 and is epidemiologically related to poultry strains (Friis et al., 2010; de Vries et al., 2015); thus, it retains pantothenate auxotrophy. There is notable differences in *Campylobacter jejuni* M1cam auxotrophy from that of the more commonly studied strain, *Campylobacter jejuni* NCTC 11168. The latter presents auxotrophy for methionine and cysteine; niacinamide, although being growth improving, is not essential and removal of pantothenate had no effect on growth in this strain (Alazzam et al., 2011). *Campylobacter jejuni* isolates 81176, on the other hand, present auxotrophy for cysteine but not methionine Vorwerk et al. (2014). Differences in substrate auxotrophy seems to be common among *Campylobacter* spp. Tenover and Patton (1987), based on 439 *Campylobacter jejuni* isolates and 46 *Campylobacter coli* isolates, have reported *Cambylobacter* auxotrophy for cysteine, cystine, arginine, proline, and methionine. The Dickgiesser and Czylwik (1985) study, based on 52 *Campylobacter jejuni* strains, reported methionine, cysteine, cystine, panthothenate, thiamine, and NAD as important substrates for auxotyping of strains.

### 4.3. Media Design

We have designed simple defined media specifically for *Campylobacter jejuni* M1cam which can support growth of 7 other *Campylobacter jejuni* strains tested in this study. The fact that previously reported MCLMAN media was designed based on *Campylobacter jejuni* NCTC11168 (Alazzam et al., 2011), which presents a different auxotrophy than *Campylobacter jejuni* M1cam, and did not include panthothenate, explains why *Campylobacter jejuni* M1cam growth was not supported in this media.

Our media, apart from *Campylobacter jejuni* M1cam specific auxotrophic substrates (methionine, pantothenate, niacinamide) and pyruvate as the carbon source, contains amino acids cysteine, serine, and glutamate/glutamine. The latter amino acids, though not essential for growth, had a significant growth promoting effect on *Campylobacter jejuni* M1cam under our experimental condition. Similar, reports have been shown for strains 81176 and NCTC11168 where substrates, though not essential, had to be added to improve growth (Alazzam et al., 2011; Vorwerk et al., 2014), although preference of these growth improving substance seems to vary between strains and culture conditions. This varying amino acid preference suggests their potential metabolic roles beyond nitrogen and the carbon source (Bièche et al., 2012). Thus, a detailed study on amino acid utilization is needed to increase our knowledge in nutrient acquisition and metabolism of *Campylobacter*. Although pyruvate transporters have not been identified, pyruvate has been shown to be used as an exogenous carbon source when present in growth medium (Mendz et al., 1997; Velayudhan and Kelly, 2002; Stahl et al., 2012; Wagley et al., 2014) and have a protective role in the presence of oxidative stress (Bolton et al., 1984; Hodge and Krieg, 1994). In our study, increasing the pyruvate concentration improved growth rate in *C. jejuni* M1cam. It should be noted that the media designed in this study was optimized to maximize the growth of *C. jejuni* M1cam. Further tests will be needed to optimize the growth of other strains, however it is clear that the designed media MM1 can support the growth of a variety of strains under different conditions.

### 4.4. Conclusion

By adopting a systems-biology approach and using a newly annotated genome of *Campylobacter jejuni* M1Cam, we have built and analyzed a strain specific genome-scale model of this organism. The analysis allowed us to identify specific auxotrophies, as well as compounds which are preferentially consumed, and this allowed us to develop a new media suitable for the study of M1Cam and other *Campylobacter* spp. strains. The model analysis also replicated the apparently paradoxical, but widely reported observation, that although *Campylobacter* spp. can operate the electron transport chain in the absence of oxygen, oxygen is nonetheless essential for growth. Experimentally confirming all aspects of our model results, in particular the generation of metabolic by-products in the media, was beyond the scope of the current contribution. However, we believe the experimental and modeling results we have presented here, when taken together, provide a solid foundation upon which to build further investigations of *Campylobacter jejuni* M1cam and related organisms.

## Data Availability Statement

The datasets generated for this study can be found in the https://data.quadram.ac.uk/dipali.singh/CJM1cam_MM/, https://data.quadram.ac.uk/dipali.singh/CJM1cam_DB/. Additionally, Pathway/Genome database generated in this study is available from Pathway Tool Registry.

## Author Contributions

NT and DS designed and supervised research plans with input from JW. DS constructed the biochemical database and genome-scale metabolic model. NT performed experiments for minimal media design with input from BP. LC performed gene predictions and annotations. ES and FN tested minimal media designed in this work in other strains. BD performed sequence analysis. MP and DS analyzed GSM and together with NT wrote the paper with revision from JW. All authors contributed to the article and approved the submitted version.

## Conflict of Interest

LC is the director of SequenceAnalysis.co.uk and was paid to perform this work. The remaining authors declare that the research was conducted in the absence of any commercial or financial relationships that could be construed as a potential conflict of interest.

## Supplementary Material

Table S1 Composition of modified DMEM/F-12 media used in this study.

Table S2 List of Gene-Protein-Reaction relationships curated in the *Campylobacter jejuni* M1cam PGDB.
