# Supplementary material for: Genome-Scale Metabolic Model Driven Design of a Defined Medium for Campylobacter jejuni M1cam
Source: Front Microbiol. 2020 Jun 19;11:1072. doi: 10.3389/fmicb.2020.01072 (PMC7318876; doi:10.3389/fmicb.2020.01072)
Supplement: Table S1 — Composition of modified DMEM/F-12 media used in this study. [file Table_1.pdf]

Table S1. Composition of initial defined media (modified DMEM/F-12*)

**INORGANIC SALTS**			
**$CaCl_2$**	793.1 µM	**NaH_2PO_4**	452.9 µM
**$Fe(NO_3)_3{\cdot}9H_2O$**	123.8 nM	**$FeSO_4{\cdot}$ $7H_2O$**	1.5 µM
**$MgSO_4$**	405.8 µM	**$CuSO_4 \cdot 5H_2O$**	5.2 nM
**KCl**	4.2 mM	**$MgCl_2$**	301.0 µM
**$NaHCO_3$**	14.3 mM	**$Na_2H_2PO_4$**	400 µM
**NaCl**	119.7 mM	**$ZnSO_4 \cdot 7H_2O$**	1.5 µM
**VITAMINS**			
**Niacinamide**	16.6 µM	**Folic Acid**	6 µM
**Biotin**	14.3 nM	**D-Calcium Pantothenate**	4.7 µM
**Thiamine HCl**	6.4 µM	**Thymidine**	1.5 µM
**Myo-inositol**	69.9 µM	**Riboflavin**	581.9 nM
**Pyridoxal**	9.8 µM	**Vit B12**	501.7 nM
**Choline chloride**	64.3 µM	**Pyridoxine HCl**	150.7 nM
**AMINO ACIDS**			
**L-Cysteine**	100.2 µM	**L- Arginine**	846.7 µM
**L-Methionine**	115.5 µM	**L-Valine**	451.3 µM
**L-Glycine**	249.8 µM	**L-Glutamic acid**	50.0 µM
**L-Asparagine**	56.8 µM	**L-Isoleucine**	415.2 µM
**L-Tryptophan**	44.2 µM	**L-Leucine**	450.1 µM
**L-Histidine**	150.2 µM	**L-Lysine**	624.1 µM
**L-Phenylalanine**	214.8 µM	**L-Proline**	149.9 µM
**L-Tyrosine**	213.6 µM	**L-Threonine**	448.8 µM
**L-Aspartic acid**	50.0 µM	**Cystine**	130.2 µM
**L-Serine**	249.8 µM	**Glutamine**	2.5 mM
**L-Alanine**	50.0 µM		
**OTHER**			
**Na Pyruvate**	0.5 mM	**Putrescine**	918.8 µM
**Hypoxanthine**	15.4 µM	**DL-68-Lipoic acid**	508.9 nM
**Linoleic acid**	149.7 nM		

*A modification of the Gibco Dulbecco's Modified Eagle Medium DMEM/F-12, no phenol red, was used as starting point to develop our minimal media for C. jejuni M1cam. No glucose was added to the formulation.
